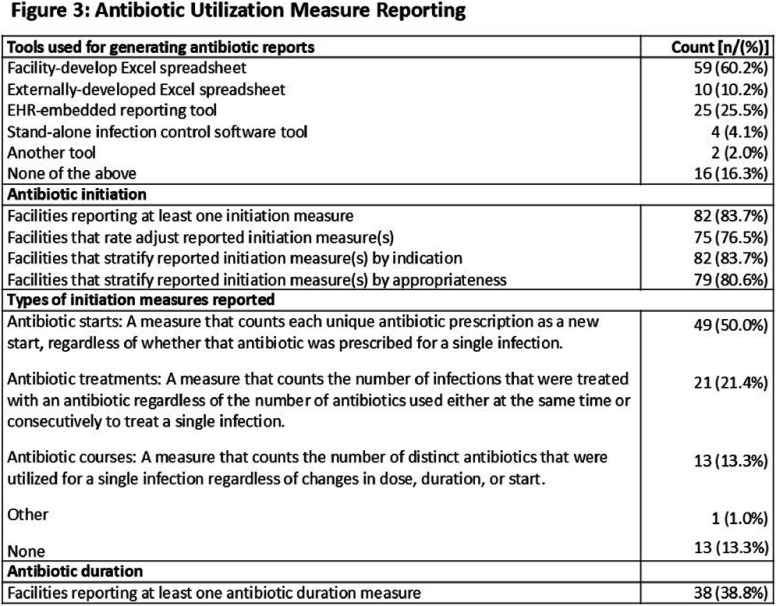# Results of a Statewide Survey of the Antibiotic Tracking and Reporting Inventory (ATARI) in Wisconsin Nursing Homes

**DOI:** 10.1017/ash.2025.257

**Published:** 2025-09-24

**Authors:** Sally Jolles, Dee Heller, Grace Multhauf, Lori Koeppel, Michele Gassman, Bikram Poudel, Madeline Langenstroer, Lindsay Taylor, Kimberly Goffard, Christinia Olivier, Mariah Welke, Christopher Crnich, James Ford

**Affiliations:** 1 University of Wisconsin Madison Department of Medicine; 2 University of Wisconsin School of Medicine; 3 UW Madison, Department of Medicine, Infectious Disease,Quality Improvement with Data Group; 4 University of Wisconsin-Madison School of Pharmacy; 5 University of Wisconsin School of Medicine and Public Health; 6 University of Wisconsin-Madison, Rocky Vista University College of Osteopathic Medicine; 7 Wisconsin Department of Health Services; 8 WI DHS, DPH, Bureau of Communicable Diseases; 9 University of Wisconsin; 10 University of Wisconsin-Madison

## Abstract

**Background:** Antibiotic tracking and reporting are core components of nursing home (NH) antibiotic stewardship programs. Nevertheless, how NHs conduct these essential activities remains poorly understood. The objectives of this study were to understand how NHs capture information on antibiotic use (AU) and characterize how AU is reported in Wisconsin NHs. **Methods:** The Antibiotic Tracking and Reporting Inventory (ATARI), a survey instrument designed to characterize the structure and process of antibiotic tracking and reporting in NHs was developed and piloted through a mixed methods approach. The instrument is organized into three sections: facility demographics, structure and process of AU data collection, and types of AU measures reported and methods of generation. After coding into REDCap, the ATARI instrument was distributed to Wisconsin NHs (n = 328) in partnership with the Wisconsin Department of Health Services. Descriptive statistics were utilized to summarize information regarding antibiotic data collection, AU reporting, and NH characteristics. **Results:** One hundred and thirty-two responses were received, of which 98 completed the instrument in its entirety for a final response rate of approximately 30%. Figure 1 details NH characteristics, including size and information system employed by responding facilities. Responding NHs reported devoting approximately 10 hours per week doing line listing activities and 18 hours per month in developing and disseminating reports (Figure 2). Paper and facility-developed Excel-based tools were used to conduct line listing activities in a majority of NHs, and 32 NHs employed more than one tool for this purpose (Figure 2). A majority, approximately 84%, of NHs reported at least one measure of antibiotic initiation although there was variation in whether facilities employed starts, courses, and treatment measures (Figure 3). Nineteen NHs utilize one or more report tools. A majority of NHs employed rate adjustment and stratification of their initiation measure by indication as well as appropriateness in their reports (Figure 3). In contrast a minority, 39%, of NHs reported a treatment duration measure (Figure 3). **Conclusions:** Wisconsin NHs devote a considerable amount of time to tracking and reporting of AU and employ a variety of low-tech tools for this purpose. There is considerable variability in the types of AU measures monitored in NHs with a majority focused on antibiotic initiation measures and lesser focus on measuring duration of therapy. These results suggest a need for standardization of AU measures in NHs as well as information systems that improve the efficiency of their collection and reporting.

**Figure:**
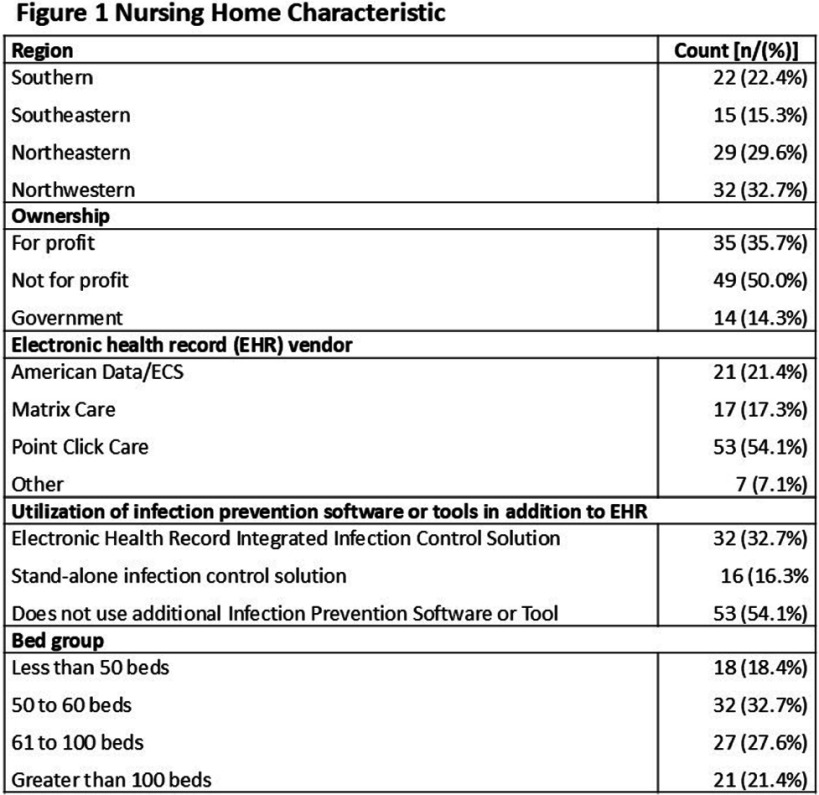

**Figure:**
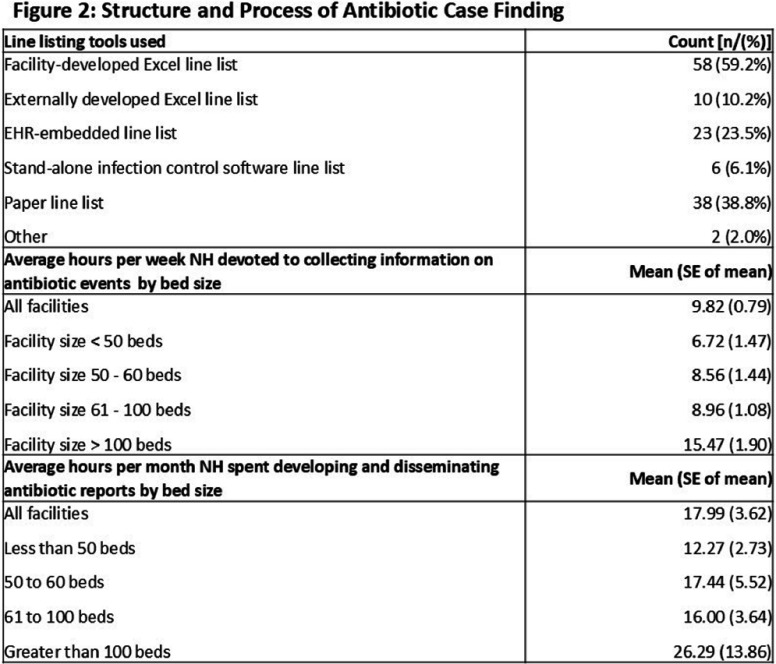

**Figure:**